# Supplementary material for: Phytochemical Composition of Different Botanical Parts of Morus Species, Health Benefits and Application in Food Industry
Source: Plants (Basel). 2022 Jan 6;11(2):152. doi: 10.3390/plants11020152 (PMC8777750; doi:10.3390/plants11020152)
Supplement: Supplementary file 1 [file plants-11-00152-s001.zip › plants-1537168-supplementary.pdf]

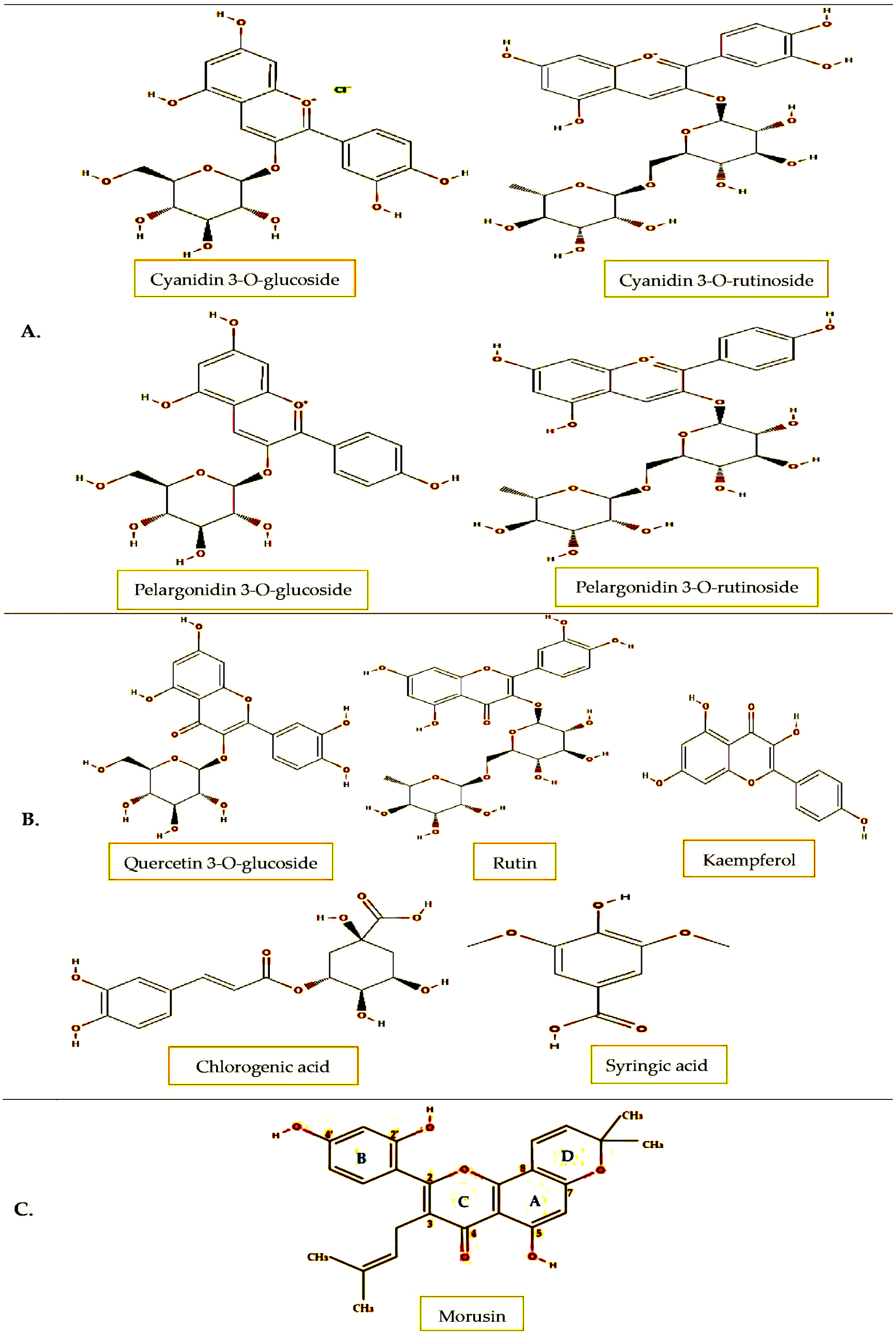

Figure S1. (**A**). The chemical structures of the four main anthocyanins identified in black mulberry fruit. (**B**). Chemical structure of the main flavonols and phenolic acids identified in mulberry's fruit and leaves. (**C**). The chemical structure of morusin. The chemical structure of phenols was generated using the molview.org program.
